# Supplementary material for: Quantifying the Effects of Combination Trastuzumab and Radiation Therapy in Human Epidermal Growth Factor Receptor 2-Positive Breast Cancer
Source: Cancers (Basel). 2022 Aug 31;14(17):4234. doi: 10.3390/cancers14174234 (PMC9454606; doi:10.3390/cancers14174234)
Supplement: Supplementary file 1 [file cancers-14-04234-s001.zip › cancers-1852933-supplementary.pdf]

cancers

MDPI

Supplementary Materials

Quantifying the effects of combination trastuzumab and radiation therapy in human epidermal growth factor receptor 2 positive breast cancer

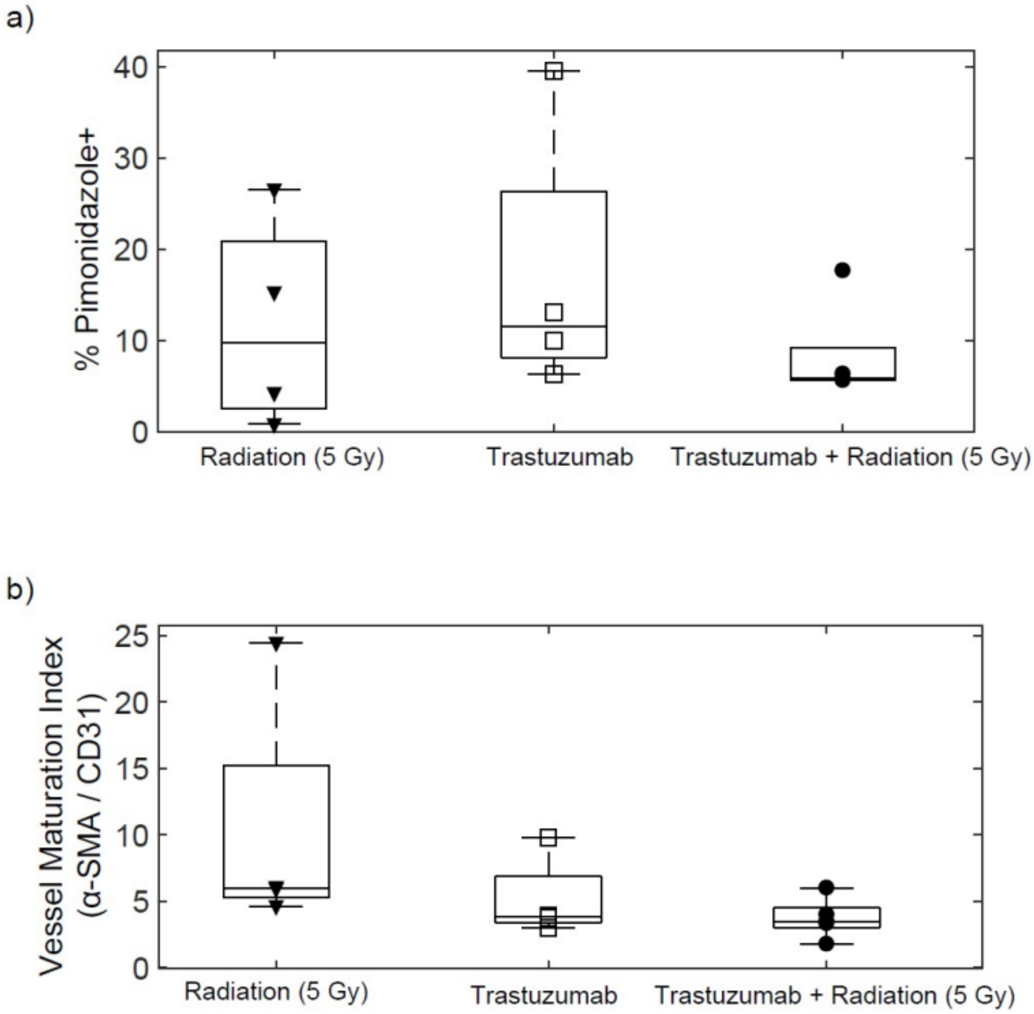

Supplementary Figure S1: Hypoxia and vascular staining in single agent radiation, trastuzumab and combination treated tumors. All radiation single agent and combination groups were treated with 5 Gy of radiation on Day 0. All trastuzumab single agent and combination groups were treated with 10 mg/kg of trastuzumab on Days 0 and 3. a) Percent pimonidazole staining revealing no significant differences between treatment groups ($P > 0.05$). B) Vascular maturation index revealing no significant differences between treatment groups ($P > 0.05$).

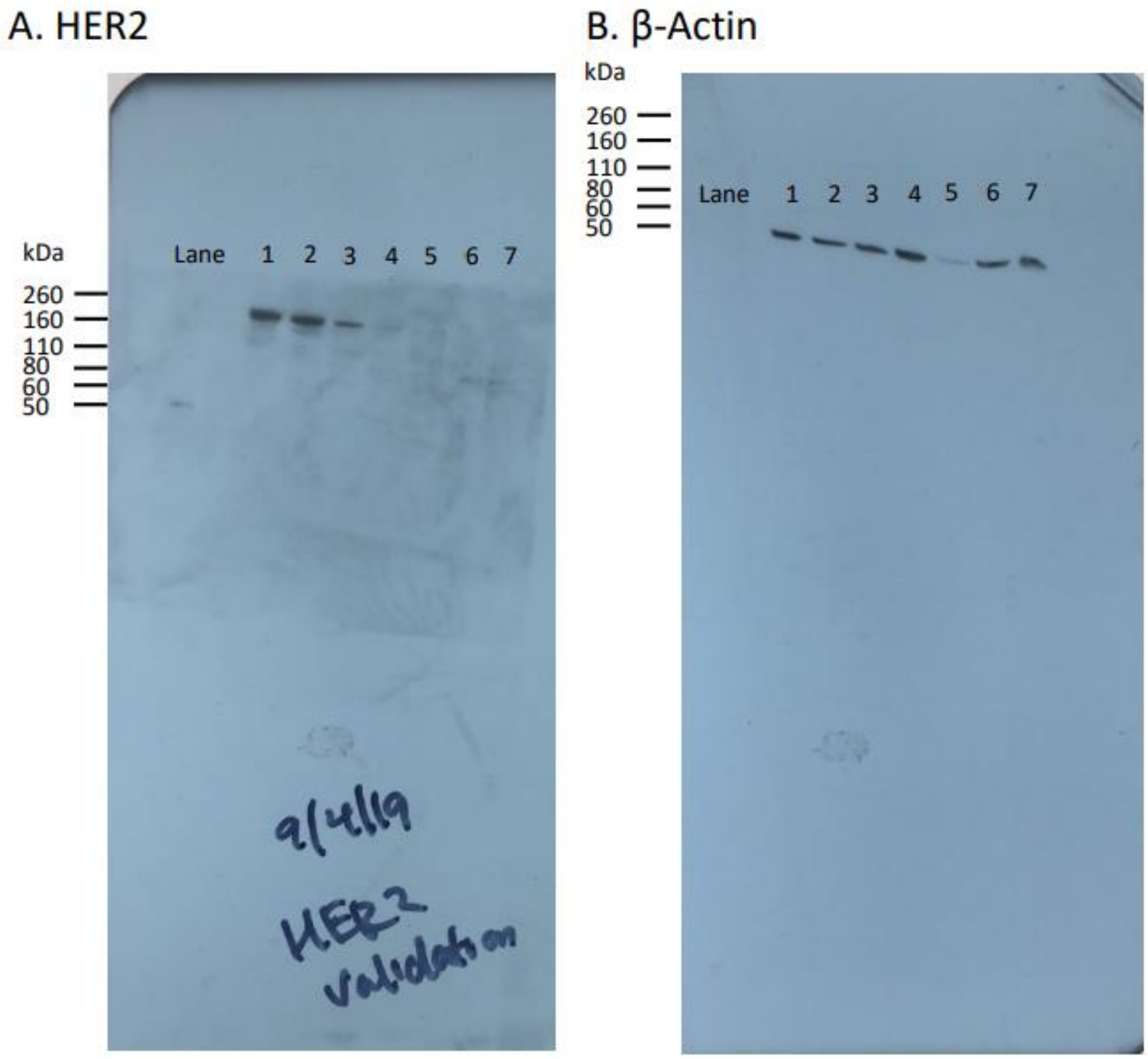

Supplementary Figure S2. Uncropped blots shown in Figure 2a of the manuscript.
